# Comparison of appropriateness of Nutri-Score and other front-of-pack nutrition labels across a group of Moroccan consumers: awareness, understanding and food choices

**DOI:** 10.1186/s13690-021-00595-3

**Published:** 2021-05-06

**Authors:** Hassan Aguenaou, Laila El Ammari, Maryam Bigdeli, Amina El Hajjab, Houria Lahmam, Saloua Labzizi, Hasnae Gamih, Anouar Talouizte, Chaimae Serbouti, Khalid El Kari, Hasnae Benkirane, Hicham El Berri, Ayoub AL-Jawaldeh, Abdelhakim Yahyane

**Affiliations:** 1 Ibn Tofail University-CNESTEN, Joint Research Unit in Nutrition and Food, RDC-Nutrition AFRA/IAEA, Rabat, 14000 Kenitra, Morocco; 2 Ministry of Health, Rabat, Morocco; 3 World Health Organisation, Rabat, Morocco; 4 Minisrty of Agriculture, Maritime Fisheries, Rural Development, Water and Forests, Rabat, Morocco; 5 World Health Organisation, Cairo, Egypt

**Keywords:** Front-of-pack nutrition labels, Objective understanding, Food choice, Perception of labels, Nutritional policy, Moroccan consumers

## Abstract

**Background:**

The front of pack nutrition label Nutri-Score, intended to help consumers orient their choices towards foods that are more favorable to health, was developed in France and applied in several European countries. Consideration is underway for its use in Morocco. This study aims to assess Moroccan consumers’ perception and objective understanding of Nutri-Score and 4 other nutritional information labels (Health Star Rating, Health warning, Reference Intakes and Multiple Trafic Light) and their impact on purchase intentions.

**Methods:**

814 participants were asked to choose among 3 food classes (yoghurts, biscuits and cold cuts), which ones they would prefer to buy among three products with different nutritional profiles and then to rank them according to their nutritional quality. Participants first performed these tasks without a visible nutritional label, and then, after being randomized to one of five labels tested, with the nutritional label visible on front of packs. Next, the full set of tested labels was presented to the participants who were asked a series of questions regarding their preferences, the attractiveness of the labels, their perceptions, intention to use and the trustworthiness placed in the labels.

**Results:**

Compared to the Reference Intake, the Nutri-Score (OR = 2.48 [1.53–4.05], *p* < 0.0001), was associated with the highest improvement in the ability to correctly classify foods based on their nutritional quality. The percentage of participants who improved their food choice was higher than those who worsened it for all the labels. For yogurts and cookies, the most significant improvements were observed for the Nutri-Score and the Reference Intakes: Concerning the perception of labels, the Nutri-Score is the label that received the highest number of positive responses, whether concerning the ease of being spotted (82.2%), of being understood (74%), and to provide rapid information (68.8%). The Nutri-Score was ranked as the preferred label by 64.9% of the participants.

**Conclusion:**

The Nutri-Score appears to be the most effective nutritional information system to inform consumers about the nutritional quality of foods in Morocco, where it could constitute a useful tool to help consumers in their food choices in situations of purchase.

## Background

In Morocco in 2017–2018, 53% of adults over 18 were overweight, including 20% obese [1]. The prevalence of obesity has increased considerably from 2007, which was 13.2% [2]. The increasing prevalence of overweight and obesity in Morocco (as in many other countries in the world) constitutes a considerable burden on public health, in particular because of the increase in chronic diseases linked to it, namely cardiovascular diseases, diabetes, cancers, respiratory pathologies, etc. Most of these chronic diseases have a multifactorial origin, including genetic determinants and environmental determinants (smoking, alcohol consumption and nutrition) [3–7]. Among the environmental determinants, an international consensus has emerged for several years on the major role that diet plays, in particular the unfavorable effects on health of excessive intakes of sugars, saturated fats, salt and insufficient intakes of fruits, vegetables and fibers, etc. [3]. However, food represents a key lever for public health policies, because it corresponds to a modifiable determinant of health that can be the subject of primary prevention interventions. Among the public health measures likely to improve diet and therefore nutritional status and health, growing interest has been shown in complementary nutritional information systems affixed to the front of food packaging (Front-Of- Pack FOP) intended to help consumers orient their choices towards foods of better nutritional quality, more favorable to health while pushing manufacturers to improve the nutritional composition of their products through reformulations [8, 9]. In Europe, FOP nutritional labels were introduced in the 1980s, first in Sweden and Denmark [10] in the 2000s in the Netherlands [11] and in the United Kingdom (Multiple Traffic Lights MTL) [12]. In 2014, Australia and New Zealand introduced the Health Star Rating system (HSR) [13], and Chile introduced health warning in 2016 [14]. Finally, in 2017, the French health authorities officially adopted the Nutri-Score [15]. Along with these government-approved programs, private companies have proposed Guidelines Dietary Amounts/Reference intakes (GDA/RIs), which were introduced in 2006 and adopted by some manufacturers in many countries [16].

In Morocco, an implementing decree on nutritional labeling was signed jointly by the Ministers of Health and Agriculture and published in the Official Bulletin on August 4, 2016 [17], with the aim of facilitating the purchase choice of consumer with regard to the nutritional composition of the products. This decree required a nutritional declaration in the form of a table specifying the content (per 100 g of food) in calories and various nutrients. This nutritional declaration includes mandatory information such as the energy value and the amount of fat, saturated fatty acids, carbohydrates, sugars, proteins and salt, as well as additional voluntary information such as monounsaturated fatty acids, polyunsaturated fatty acids, polyols, starch, fiber, vitamins and minerals. However, to be effective, nutrition labeling must be accessible to all consumers, and the nutrition declaration is difficult for consumers to use. It appears on the back of the packaging and its interpretation is difficult for the vast majority of the Moroccan population. Considering the public health challenges, discussions are underway to set up a nutritional system complementary to the nutritional declaration in the form of a nutritional label intended to be affixed on the front of food packaging which is simple and intuitive and understandable by all, allowing Moroccan consumers at a glance to have an idea of the nutritional quality of foods at the time of purchase. This discussion is part of the implementation of the Moroccan National Nutrition Program which is aligned with the orientations of the multisectoral strategy for the prevention and control of non-communicable diseases (2019–2029), the Obesity Operational Plan and the policy of the Ministry of Health in the fight against maternal and infant mortality and morbidity.

In this context, particular interest has been paid to Nutri-Score, which is based on solid scientific background that have validated its underlying algorithm and its graphic format [18]. However, whether the graphic format of the Nutri-Score developed in France is applicable in the context of other countries, its efficiency in Morocco needs to be investigated. The Nutri-Score has been the subject of studies in several countries covering different cultural and dietary contexts. A recent study undertaken in twelve different countries from different continents (France, Spain, Germany, UK, Denmark, Bulgaria, USA, Canada, Mexico, Argentina, Australia, Singapore) assessed the ability of five nutritional labels, including HSR, MTL, Nutriscore, RIs and Warnings to help consumers judge the nutritional quality of different foods and help them guide their choices [19]. Out of all 12 countries studied, the Nutri-Score appears to be the most effective in helping consumers to judge, on a relative basis, the nutritional quality of foods. Several studies have also shown that the Nutri-Score has a high capacity to discriminate foods based on their nutritional composition, with similar trends in all countries, and good consistency with nutritional recommendations [20, 21]. However, no study has been carried out in the context of the Maghreb, and in particular in Morocco.

The objective of the present study supported by the Ministry of Health and by the WHO is to examine the perception and objective understanding of 5 labels of nutritional information used in the world (including Nutri-Score) in a group of Moroccan consumers and their impact on their purchasing intentions.

## Materials and methods

The study was conducted from (October 2019 to December 2019) followed the methodology described in the framework of the international FOP-ICE study published by Egnell and al in 2018 [19], by adapting it to the Moroccan context for data collection and products tested.

### Population

The study involved 814 participants recruited using quotas on gender (half male and female) and age: 20% of adolescents aged 10 to 18 years (50% girls and 50% boys), 80% of adults over 18 years, 3 age groups, 18–30 years / 31–50 years / 51 years and over (50% men and 50% women) and socio-economic status quotas (low, medium, high). Participants were recruited from various supermarkets chains (Marjane, Acima, Carrefour, Aswak salam, Bim and various local shops) as clients as well as medical consultation offices, and from various stakeholders in the study, who each selected people while respecting the predefined quotas.

Neighborhoods were selected (three types – one third per type: low, middle and upper class) to guarantee the heterogeneity of socio-economic variables based on the survey frame of the 2014 HCP census. The study took place in 5 regions of Morocco: Fes / Meknes; Marrakech / Safi; Casa / Settat; Rabat salé / Kenitra; Souss / Massa. One hundred participants were selected according to the predefined quota in each region excepted in the Souss / Massa region, where it was possible to collect only 47 questionnaires due to technical problems.

### Ethical considerations

The study protocol was approved by the Ethics Board of the Faculty of Medecine and Pharmacy, Mohammed University in Rabat – Morocco. (Ethical Approval number 69 delivered on 31 January 2017). The project was also validated by the scientific, technical and advisory committee on nutrition (institutionalized by a decision of the Ministry of Health in 2018).

Before data collection by questionnaire, invited participants were informed about the study objectives and methods and signed an informed consent form.

### Data collection methods

The study data was either collected by questionnaires in face-to-face interviews with investigators or via the Internet using an online questionnaire:

- 447 participants responded to investigators who were initially trained in a standardized way at the Regional Designated Center for Nutrition (RDC-Nutrition, Ibn Tofaïl- University-CNESTEN). The questionnaire was pre-tested for verification before generalization. The questionnaire was distributed in two languages: Arabic and French. Randomization was used to allow all labels to be tested equally.nnaire.
- 367 participants answered an online questionnaire via the survey software “Survey Monkey”. Entering the questionnaire into the software (in two languages Arabic and French) was performeèd by applying bulk randomization to allow all labels to be tested.

### Nutritional labels tested

**Fig. 1:**
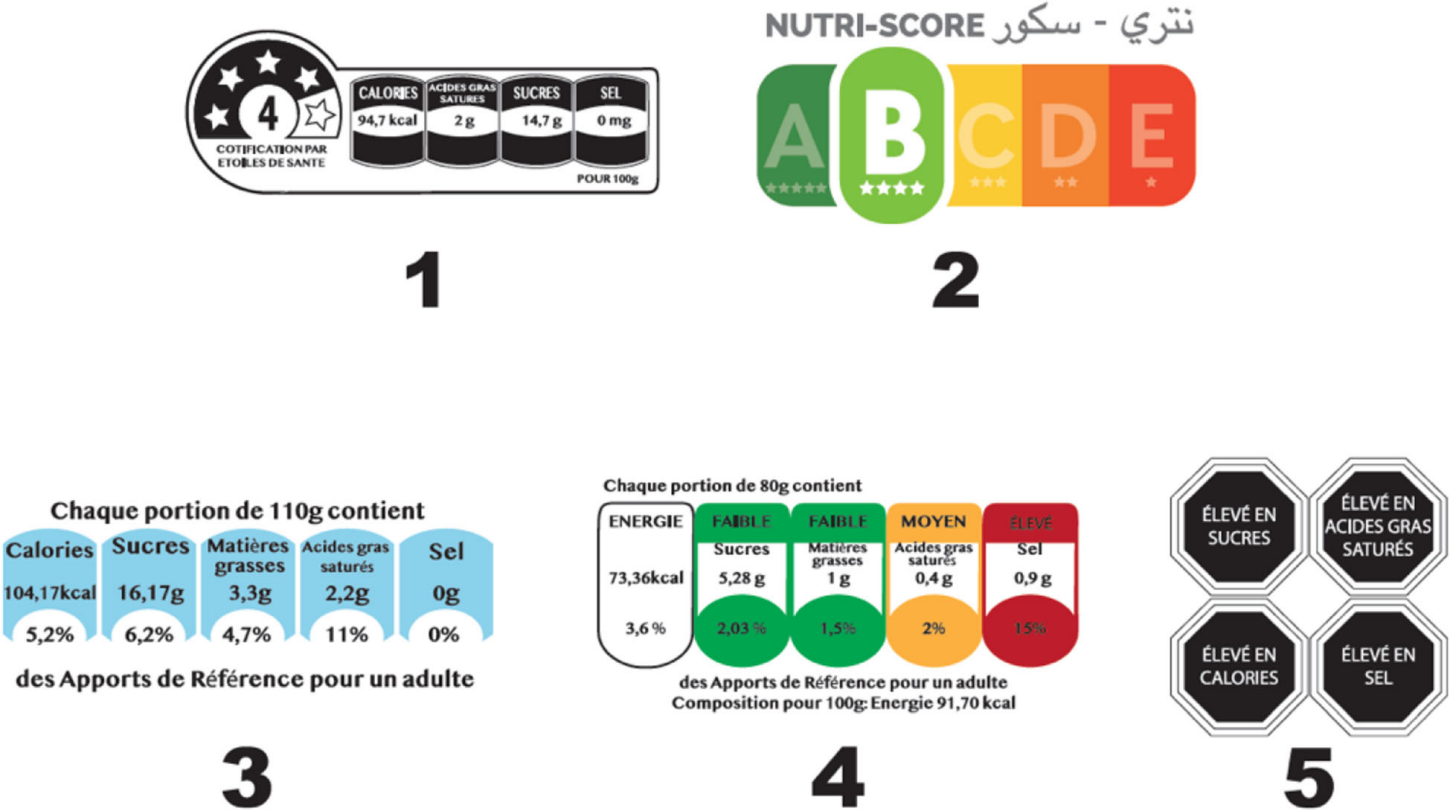
The five labels studied: (1) Health Star Rating; (2) Nutri-Score; (3) Reference Intakes; (4) Multiple Trafic Light; (5) Warning Symbol. Comparison study of the relevance of Nutri-Score and other nutritional labels on the front of the packaging. Morocco (2019)

Five nutritional labels were tested (Fig. 1): 1. Reference Intakes (Reference intakes implemented by certain manufacturers in different countries since 2006), 2. Warnings (Health warning symbol implemented in Chile since 2016), 3. Nutri-Score (adopted in France since 2017, and since in Belgium, Spain, Germany, the Netherlands, Luxembourg and Switzerland), 4.Health Star Rating, HSR (System of classification of health stars: implemented in Australia and New Zealand since 2014) and 5. Multiple Trafic Light (MTL, Multiple Traffic Lights implemented in the UK since 2005) Fig. 1.

### Choice of products

**Fig. 2:**
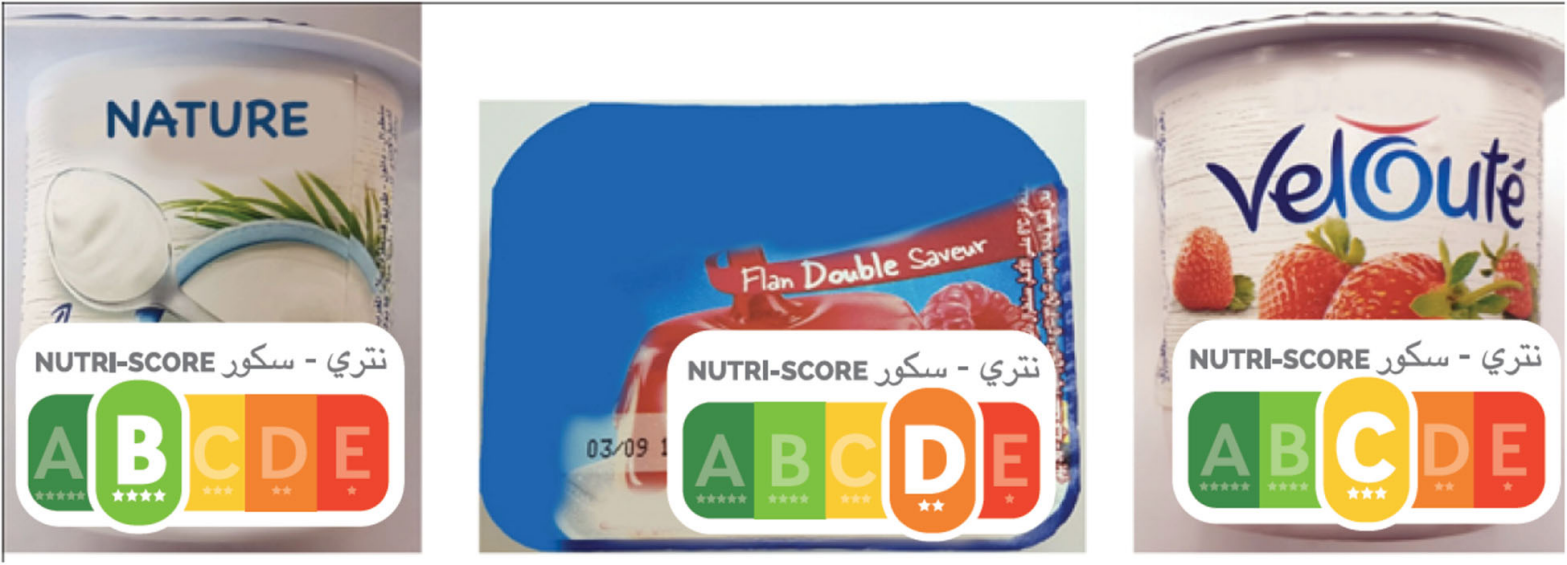
Example of a product with the label on the front presented in the questionnary: Comparison study of the relevance of Nutri-Score and other nutritional labels on the front of the packaging. Morocco (2019)

Three product categories (yogurt, biscuit and cold cuts) were tested in the present study. This choice was based on the fact that these are processed foods among the most consumed by the Moroccan population [22] and corresponding to products whose nutritional composition varies greatly. Within each food category, a set of three products with distinct nutrient profiles (high, medium and low quality) were selected, which allowed the products to be classified according to their nutritional quality. The labels were affixed in the same place on each food product and covered the same area on the packaging (Fig. 2). To avoid unduly influencing participants’ perceptions of food products, no other nutritional information or quality indicators were provided.

### Procedure

First, the questionnaire collected information on the participants’ gender, age, income, level of education, occupation, involvement in shopping, place of residence, city of residence, marital status, self-estimation of the level of nutritional knowledge.

Secondly, the questionnaire assessed the food choices, the objective understanding of the labels and their perception by the participants. To prevent participants from getting used to paying special attention to nutritional labels, the study was conducted by testing food choices first, then objective understanding and finally perception. Participants were first exposed to three sets of products (three types yogurts, biscuits and cold cuts) without labels on the front of the packages. Participants were asked to designate the three products they would buy, with an option “I would not buy any of these products”. After the choice tasks, participants were asked to rank all three products according to their nutritional quality (1- Best nutritional quality, 2- Intermediate nutritional quality and 3- Worst nutritional quality), with an “don’t know” option also available. The selection and ranking phases were carried out by food category, successively, and the order of presentation of the food categories was randomized among the respondents.

Thirdly, all the labels were presented to the participants who were questioned on a series of questions concerning their preferences, the attractiveness of the labels, their perceptions, the conditions of use and the trust attributed to the labels.

### Data analysis

A score between 1 and 3 points was awarded for the choice of each food category, with + 1 for the product of the lowest nutritional quality, + 2 for the intermediate nutritional product, and + 3 points for the product of the highest nutritional quality, this operation was done first without label and then with label. No points were awarded when participants selected the option “I will not buy any of these products”.

A score was then calculated for each food category using the point difference between the answer with and without a label, giving a discrete score ranging from − 2 to + 2 points. Finally, an overall score was calculated by adding the score for each category, resulting in a score between − 6 and + 6 points for each participant.

The percentage of participants whose food choices deteriorated or improved between the response with and without a label were calculated for each label and by food category.

A multivariate ordinal logistic regression model was performed to measure the association between the choice score and the type of label.

For the participants’ ability to correctly classify the products in each category based on their nutritional quality, the answer was considered correct when all three products in the category were correctly classified, leading to a score of + 1 point for each category of food, while 0 points were awarded for incorrect classification. Thus, for each food category, a grading accuracy score was calculated using the point difference between responses with and without label, ranging from − 1 to + 1 points and 0 indicating no change; And leading to an overall score of between − 3 and + 3 points for each participant.

The percentage of correct answers with and without a label were calculated for each label and by food category.

A multivariate ordinal logistic regression model was performed to measure the association between choice score and label type. Given the lack of effect of the “Reference Intakes” label reported in the literature (Egnell et al., 2018a), this label was used as a reference category in the ordinal logistic regression of the models.

For the analysis of choice and classification of products, sex, place of residence, marital status, age, level of education, level of income, knowledge of nutrition, self-assessment of food, profession, responsibility for shopping were introduced as covariates.

All variables showing statistical significance at the *p* < 0.25 level in bivariate models were included in the model.

The perception of labels was represented by the percentage of positive or negative appreciations made by the participants.

## Results

A total of 814 participants completed the questionnaire. The characteristics of the sample are shown in Table 1. 40.2% were men and 59.8% were women; 15.8% were aged 10–17 years, 30% were adults aged 18–29 y, 33.1% were aged 30–49 y and 21.1% were over 50. Among adults (671) 66.5% had a university education level, 28.5% a secondary level and 5% a primary level or Msid (The Koranic school or the Msid is a religious institution in almost all the cities and the companions of the Moslem countries and towards which the kids flock in order to learn the Koran, the Arabic language, mathematics ...); 24.1% were civil functionary in the public sector, 24.1% work in the liberal sector, 7.2% were housewives, 34.6% were students or pupils and 4.2% were unemployed. Only two thirds of the population surveyed agreed to declare their income: 16.2% earned less than 3000 dhs (330 US dollars) per month; 10.4% between 3000 and 5999 dhs (330 and 660 US dollars); 12.3% between 6000 and 9999 dhs (660 and 1100 US dollars): 9.7% between 10,000 and 15,999 dhs (1100 and 1760 US dollars) at 9.7%, and only 5.6% of the participants declared having an income in excess of the 25,000 dhs (2750 US dollars) per month. Knowing that, according to the report of the High Commission for Planning 2020, the poverty rate was 4.8% at the national level.

**Table 1:** Characteristics of the study population: Comparison study of the relevance of Nutri-Score and other nutritional labels on the front of the packaging. Morocco (2019)

		Number	(%)
**Gender**	Men	327	40,2%
	Women	487	59,8%
**Place of residence**	Urban	772	95,2%
	Rural	39	4,8%
**Age (years)**	10–18	126	15,8%
	18–29	239	30,0%
	30–49	264	33,1%
	> 50	168	21,1%
**Marital status**	Single	398	49,1%
	Married	364	44,9%
	Divorced	33	4,1%
	Widowed	16	2,0%
**Highest qualification**	Primary or Msid	41	5,0%
	Secondary	232	28,5%
	University	540	66,5%
**Profession**	State employee	195	24,1%
	Private sector employee	120	14,8%
	Liberal profession or business manager	75	9,3%
	Manual worker	29	3,6%
	Retired	18	2,2%
	Housewife	58	7,2%
	Student	280	34,6%
	Without profession	34	4,2%
**Monthly income level (DHS)**	< 3000 DH	118	16,2%
	3000–5999 DH	76	10,4%
	6000–9999 DH	90	12,3%
	10,000–15,999 DH	71	9,7%
	16,000–25,000 DH	66	9,1%
	> 25,000 DH	41	5,6%
	I don’t know	132	18,1%
	I refuse to answer	135	18,5%
**Purshasing manager for the home**	Yes	315	39,2%
	No	302	37,6%
	Purchases are distributed fairly throughout the household	187	23,3%
**Perception of the participant’s diet**	I have a very balanced diet	47	5,8%
	I have a balanced diet	308	38,0%
	I have an unbalanced diet	338	41,7%
	I have a very unbalanced diet	118	14,5%
**Nutritional knowledge**	I know a lot about nutrition	147	18,1%
	I know enough about nutrition	367	45,1%
	I know a little about nutrition	222	27,3%
	I don’t know anything about nutrition	78	9,6%
**Yoghurt purchase frequency**	Always	255	31,4%
	Often	255	31,4%
	Sometimes	212	26,1%
	Rarely	74	9,1%
	Never	16	2,0%
**Cold cuts purchase frequency**	Always	106	13,2%
	Often	132	16,4%
	Sometimes	205	25,4%
	Rarely	216	26,8%
	Never	147	18,2%
**Biscuits purchase frequency**	Always	71	8,8%
	Often	123	15,2%
	Sometimes	226	27,9%
	Rarely	261	32,2%
	Never	130	16,0%

Among the participants surveyed, 18.1% declared having good knowledge in nutrition, 45.1% having average knowledge in this field while 36.9% declared having little or no knowledge in the matter. The analysis of the frequency of purchase of the 3 categories of food products concerned by the study showed that yogurt was bought often and / or always by 62.8% of participants, cold meats were bought often and / or always by 29.6%, while 24% of participants often and / or always bought cookies.

**Fig. 3:**
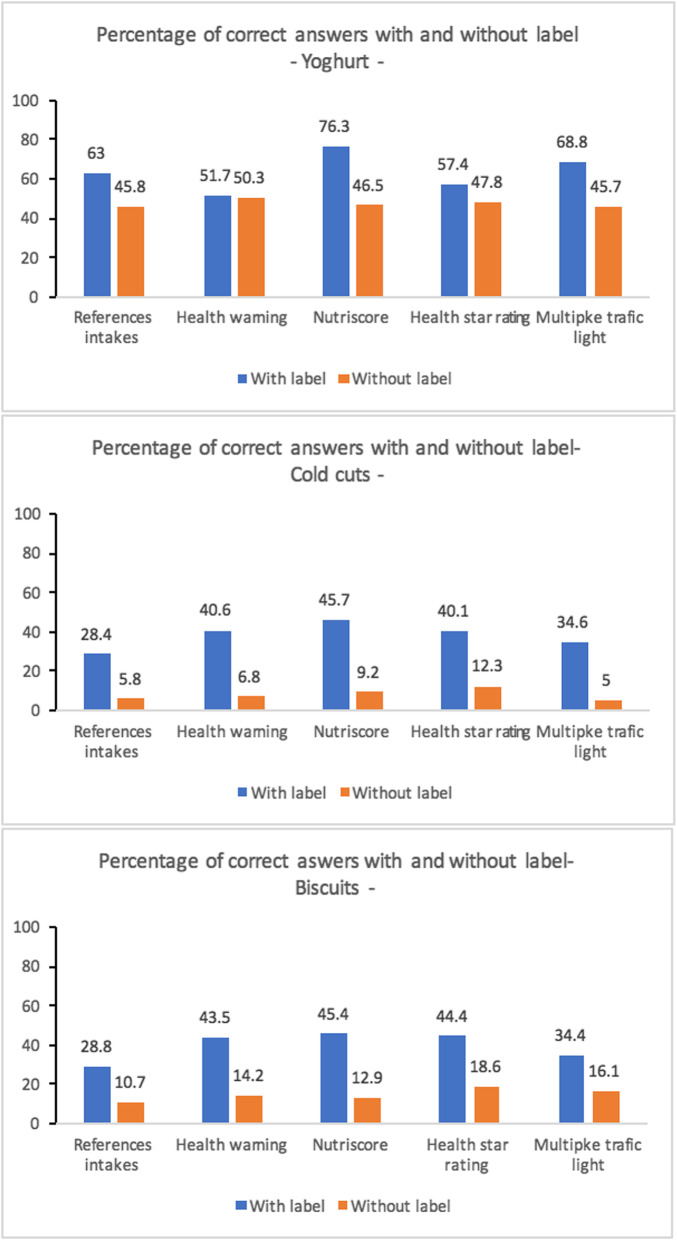
Comparison of correct answers for ranking products according to nutritional quality with and without label: Comparison study of the relevance of Nutri-Score and other nutritional labels on the front of the packaging. Morocco (2019)

Regarding objective understanding, that is, the ability of labels to help consumers correctly classify the nutritional quality of foods, compared to the assessment phase without a label, the addition of labels on product packaging improved the proportion of correct responses of the study population (Fig. 3). However, all the labels did not have the same impact: the Nutri-Score improved responses by more than 30% for all food categories tested (76.3% with label against 46.5% without label for yogurt, 45.7% vs. 9.2% for cold cuts and 45.4% vs. 12.9% for cookies), followed by the Health Star Rating (57.4% vs. 47.8% for yogurt, 40.1% vs. 12.3% for cold cuts, 44.4% vs. 18.6% for cookies), Multiple Traffic light (68.8% against 45.7% for yogurt, 34.6% against 5% for cold cuts, 34.4% against 16.1% for cookies), then the Health warning (51.7% against 50.3% for yoghurt, 40.6% against 6.8% for cold cuts, 43.5% against 14.2% for cookies). Finally, the References intake label showed the smallest increase in the number of correct answers (63% against 45.8% for yogurt, 28.4% against 5.8% for cold meats, 28.8% against 10.7% for cookies).

The Nutri-Score was associated with the greatest improvement in the ability to correctly classify the nutritional quality of products (Odds Ratio [95% confidence interval]: OR = 2.48 [1.53–4.05], *p* < 0.0001), followed by MTL (OR = 1.51 [0.93, 2.46], *p* = 0.1), Health Warnings (OR = 1.43 [0.87, 2.35], p = 0,2), and the Health star rating (OR = 1.17 [0.72, 1.89], *p* = 0.5) (Table 2). The effect of the labels appeared slightly more effective in the cold cuts products category compared to the other two categories.

**Table 2:** Associations between label and the ability to correctly rank products according to nutritional quality, by label and food category^a^: Comparison study of the relevance of Nutri-Score and other nutritional labels on the front of the packaging. Morocco (2019)

Product classification			
Yoghurt	OR	95% IC	***P***-value
*Health warning*	0.60	0.34, 1.04	0.072
*Nutriscore*	1.95	1.15, 3.33	0.014
*Health star rating*	0.85	0.50, 1.46	0.6
*Multiple Traffic Light*	1.46	0.86, 2.50	0.2
**Cold cuts**			
*Health warning*	2.13	1.19, 3.84	0.012
*Nutriscore*	2.69	1.52, 4.80	< 0.001
*Health star rating*	1.53	0.86, 2.71	0.15
*Multiple Traffic Light*	1.69	0.95, 3.03	0.078
**Biscuits**			
*Health warning*	1.90	1.04, 3.48	0.038
*Nutriscore*	2.57	1.44, 4.64	0.001
*Health star rating*	1.60	0.89, 2.89	0.12
*Multiple Traffic Light*	1.09	0.59, 2.02	0.8
**All products**			
*Health warning*	1.43	0.87, 2.35	0.2
*Nutriscore*	2.48	1.53, 4.05	< 0.001
*Health star rating*	1.17	0.72, 1.89	0.5
*Multiple Traffic Light*	1.51	0.93, 2.46	0.10

^a^ The reference for multivariate ordinal logistic regression was the “Reference Intakes” label. The multivariate model was adjusted for sex, place of residence, marital status, age, level of education, level of income, occupation, responsibility for shopping. OR: Odds Ratio; CI: confidence interval

**Fig. 4:**
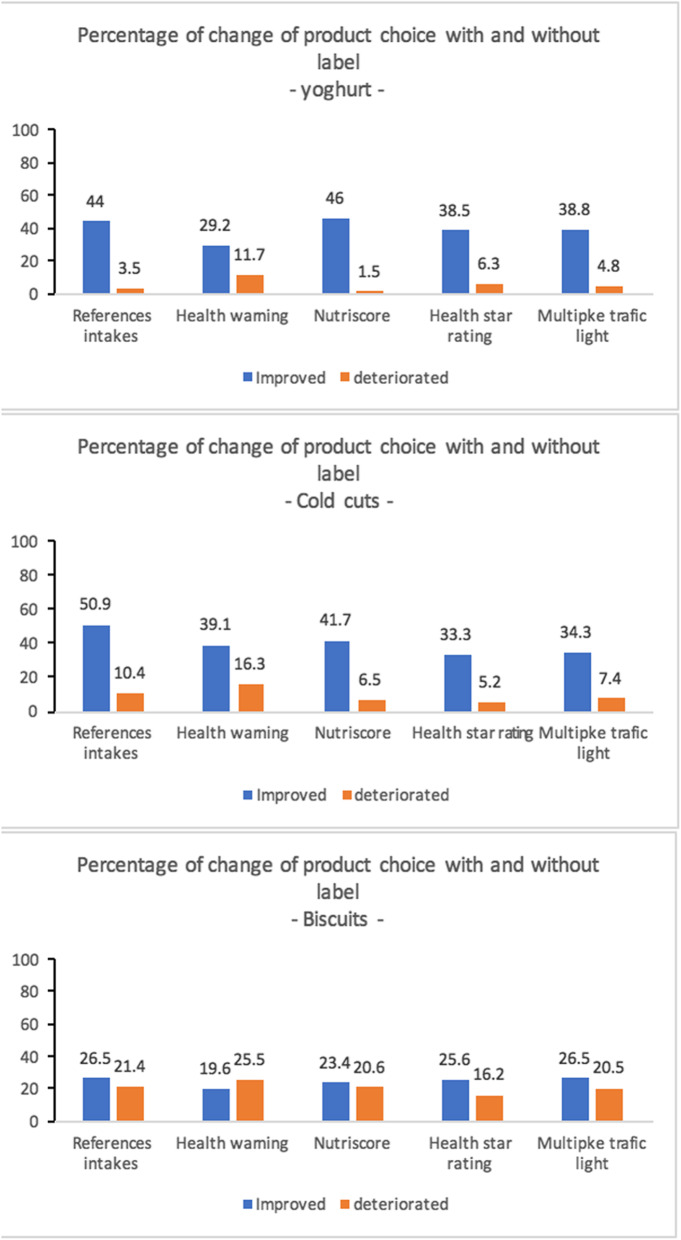
Change in product choice with and without labels: Comparison study of the relevance of Nutri-Score and other nutritional labels on the front of the packaging. Morocco (2019)

Regarding the food choices declared (Fig. 4), the percentage of people who improved their food choice (choice of a product of better nutritional quality) for all the labels was higher than those who deteriorated their choice (respectively by 19.6 to 50.9% versus 1.5 to 25.5%). This improvement varied according to the products: the improvement was more marked with yoghurts, followed by cookies and finally cold meats. For yoghurts and cookies, the most significant improvements were observed for Nutri-Score and Reference Intakes).

The results of the ordinal logistic regression models are shown in Table 3. Compared to the “Reference Intakes” label, no significant association was found between the labels and the change in the nutritional quality of food choices, globally or by product category, with the exception of the “Health warning” which deteriorated the choice of participants for the purchase of yoghurt.

**Table 3:** Associations between labels and change in nutritional quality of food choices, by label and food category in participants who made a choice^a^: Comparison study of the relevance of Nutri-Score and other nutritional labels on the front of the packaging. Morocco (2019)

Product choice			
Yoghurt	OR	95% CI	P-value
*Health warning*	0.47	0.27, 0.80	0.006
*Nutriscore*	1.05	0.63, 1.75	0.8
*Health star rating*	0.75	0.46, 1.24	0.3
*Multiple Traffic Light*	0.76	0.46, 1.27	0.3
**Cold cuts**			
*Health warning*	0.66	0.36, 1.18	0.2
*Nutriscore*	1.07	0.60, 1.88	0.8
*Health star rating*	1.14	0.66, 1.98	0.6
*Multiple Traffic Light*	0.94	0.54, 1.64	0.8
**Biscuits**			
*Health warning*	0.65	0.35, 1.20	0.2
*Nutriscore*	0.86	0.50, 1.51	0.6
*Health star rating*	0.77	0.43, 1.39	0.4
*Multiple Traffic Light*	0.62	0.35, 1.12	0.12
**All products**			
*Health warning*	0.55	0.29, 1.06	0.072
*Nutriscore*	0.97	0.53, 1.78	> 0.9
*Health star rating*	1.01	0.55, 1.86	> 0.9
*Multiple Traffic Light*	0.65	0.35, 1.19	0.2

^a^ The reference for multivariate ordinal logistic regression was the “Reference Intakes” label. The multivariate model was adjusted for gender, place of residence, marital status, age, level of education, level of income, occupation, responsibility for shopping. OR: Odds Ratio; CI: confidence interval

Regarding the results on the perception of labels, (Tables 4 and 5), overall, the Nutri-Score appeared to be the label that received the highest number of positive responses regarding the ease of being spotted (82, 2%), to be understood (74%), and to provide rapid information (68.8%). In addition, the Nutri-Score was ranked as the preferred label among 64.9% of the participants, while only 7.1% of the participants ranked it as the least preferred. MTL came in second position: 16.8% of participants declared that it helped to choose better products for health, also providing reliable information (21.8%), however, respectively 8.4 and 9.4% declared that it was not a label that was easy to understand and locate. Then, 18.8% of participants declared that RI provided reliable information quickly, and 21.6% declared it gave them the information they need, in contrast, a third of the population qualified it as long to understand. Finally, the least preferred label of the participants was the Health Warning (47.7%), considered as guilt creating by 42.9% of the participants.

**Table 4:** Distribution of participants according to the positive appreciation of the labels: Comparison study of the relevance of Nutri-Score and other nutritional labels on the front of the packaging. Morocco (2019)

	Reference Intakes	Health warning	Nutriscore	Health star rating	Multiple Traffic Light
**This is my favourite label**	107 14.3%	20 2.7%	487 64.9%	39 5.2%	97 12.9%
**This label helps quickly choosing better products for my health**	112 14.9%	31 4.1%	437 58.2%	45 6%	126 16.8%
**This label is easy to understand quickly**	77 10.2%	27 3.6%	552 74%	32 4.3%	70 9.4%
**This label is easy to spot quickly**	53 7%	14 1.9%	611 82.2%	12 1.6%	63 8.4%
**This label gives me the information I need**	161 21.6%	32 4.3%	337 45.2%	46 6.2%	170 22.8%
**This label inspires me confidence**	138 18.6%	25 3.4%	391 52.6%	57 7.7%	132 17.8%
**This label allows me to have reliable information quickly**	140 18.8%	26 3.5%	355 47.6%	62 8.3%	163 21.8%
**This label provides quick information**	86 11.4%	27 3.6%	519 68.8%	35 4.6%	87 11.5%
**I want it to be present on the packaging**	103 13.7%	19 2.5%	490 65.2%	44 5.9%	95 12.6%

**Table 5:** Breakdown of consumers according to negative appreciation of labels: Comparison study of the relevance of Nutri-Score and other nutritional labels on the front of the packaging. Morocco (2019)

	Reference Intakes	Health warning	Nutriscore	Health star rating	Multiple Traffic Light
**This is the label I like the least**	140 18.5%	349 47.7%	53 7.1%	125 16.7%	88 11.7%
**This label is guilt-creating**	124 16.9%	321 42.9%	75 10%	85 11.6%	127 17.3%
**This label is too complicated to understand**	191 25.5%	224 29.9%	72 9.6%	150 20%	112 15%
**This label takes too long to understand**	252 33.6%	108 14.4%	5.6%	133 17.8%	214 28.6%

## Discussion

In the present study performed on a group of Morrocan consumers, compared to the the RIs, the Nutri-Score was the nutrition label that produced the largest increase in participants’ ability to correctly rank the nutritional quality of products, followed by the MTL and other nutritional labels currently used in various countries. The Nutri-Score appeared to be the one with the best performance in helping participants to understand the nutritional quality of food products and to orient their choices towards foods more favorable to health. These results are consistent with previous studies developed in France [23] and with the overall results of the FOP-ICE study carried out in 12 countries around the world including european, north- and south-american, asian and oceanian countries [19] and the specific study carried out in 12 European countries [24].

The Nutri-Score was also ranked by the participants as the preferred label and considered as the easiest of being spotted, to be understood and to provide rapid information. These results are also consistent with studies showing that Nutri-Score is strongly supported by consumers and appears as the preferred format compared to other labels especially by populations with the lowest levels of nutritional knowledge [25–27].

In addition to its synthetic character, the graphic characteristics of Nutri-Score may explain the better performance observed in the participants to our study, and more particularly the use of coding using intuitive colors ranging from green to red. The presence of the color code could be effective in drawing attention to the label and making it easier to understand [28]. The use of colors from green to red is particularly important because the human eye is biologically adapted to identify these colors well and quickly due to the specificity of color recognition in the retina [29]. In addition, green and red are easily interpreted due to the analogy with traffic lights known to all of the general public.

But beyond the use of colors, a key aspect of the better performance of the Nutri-Score as we observed in our study as in many other studies [18] could be linked to its synthetic character providing consumers with information on overall nutritional quality in the form of a single indicator summarizing the nutritional composition of foods, rather than providing multiple information on different nutritional elements. Previous studies have found that consumers more easily understand synthetic indicators [30–32], especially vulnerable populations, which are an important target for nutrition policies, especially in Morocco. Given the very limited time during which decisions are made in purchasing situations [30], the use of an overall synthetic indicator, such as Nutri-Score, may also provide an advantage due to the reduced cognitive workload that it requires for its interpretation [25, 33]. On the other hand, the use of a synthetic indicator rather than by nutrient can also reduce confusion related to the interpretation of nutritional terms (e.g. saturated fatty acids, sugars, sodium) and facilitate the comparison of the nutritional quality of food products [34]. Finally, the fact of being based on colors makes it understandable by everyone, even people who cannot read. Altogether, the better perception and performance of Nutri-Score with regard to objective consumer understanding can be related to the combination of the use of intuitive colors and a simple summary format, with a gradual format that seems understandable to all.

All these points are particularly important for the choice of an efficient front-of-pack nutritional label in a country like Morocco.

The Nutri-Score therefore appears useful in raising awareness of nutrition among Moroccan consumers, improving their understanding of the nutritional quality of foods, stimulating the purchase of healthier foods and having an impact on the nutritional quality of food.

The strengths of this study include the sample size and the fact that the protocol uses sets of three food items (rather than the use of sets of two foods, as is often the case in other studies), which makes it possible to approach realistic situations while reducing the risk of correct answers simply linked to chance. In addition, the tested foods were selected from foods usually consumed by the Moroccan population and to provide a clear nutritional difference between the products that can be objectified by the different nutritional labels to facilitate the classification process. Finally, a potential learning effect was also controlled by randomizing the order of presentation within sets and between food categories.

One of main the limits of this study is the method of recruiting subjects, done on a voluntary basis using quotas and not a representative sample of the population. Due to the recruitment method and the questionnaire collection method, the most disadvantaged populations are not or little represented and we have an over-representation of educated populations. Caution should therefore be exercised in extrapolating the results to the general Moroccan population. Even if it was demonstrated in studies performed in France [32, 35, 36] that the effect of Nutri-Score was particularly clear in disadvantaged populations (subjects with low socio-economic level, lower educational level and lower nutrition knowledge) further studies are needed to confirm the potential beneficial effects of Nutri-Score on vulnerable populations in different countries.

## Conclusion

A nutritional label on the front of food packaging is seen by WHO as a promising (best-buy) strategy to fight chronic nutrition-related diseases in all countries. The results of our study performed on a group Moroccan consumers and data from work carried out in other contexts suggest that among the options available, the Nutri-Score appears to be the most effective nutritional information system to inform consumers about the nutritional quality of food and could constitute for Morocco a useful tool to help consumers consumers in their food choices in their purchasing situations. It could also be an incentive criterion for manufacturers to reformulate their products, which could lead to a general improvement in the food supply for the Moroccan population. The implementation of the Nutri-score must be accompanied by an adapted communication campaign explaining its use and its interest.

## Data Availability

The raw data supporting the conclusion of this article will be made available by the authors, without undue reservation after terminating its exploitation for future publications.

## Abbreviations

FOP: Front-Of- Pack
OR: Odds Ratio
UK: United Kingdom
MTL: Multiple Traffic Lights
HSR: Health Star Rating system
GDA: Guidelines Dietary Amounts
RIs: Reference intakes
WHO: World Health Organisation
ICE: International Comparative Experimental
HCP: High Commission for Planning
CNESTEN: National Energy Center of Nuclear Sciences and Techniques
DHS: Dirhams
US: United States
IC: Confidence Interval
